# A novel point-of-care system for high-speed real-time polymerase chain reaction testing for epidermal growth factor receptor mutations in bronchial lavage fluids after transbronchial biopsy in patients with non-small cell lung cancer

**DOI:** 10.3892/ijo.2015.2875

**Published:** 2015-02-04

**Authors:** TOMOHIRO SAKAMOTO, MASAHIRO KODANI, MIYAKO TAKATA, HIROKI CHIKUMI, MASAKI NAKAMOTO, SHIZUKA NISHII-ITO, YASUTO UEDA, HIROKI IZUMI, HARUHIKO MAKINO, HIROKAZU TOUGE, KENICHI TAKEDA, AKIRA YAMASAKI, MASAAKI YANAI, NATSUMI TANAKA, TADASHI IGISHI, EIJI SHIMIZU

**Affiliations:** Division of Medical Oncology and Molecular Respirology, Faculty of Medicine, Tottori University, Yonago, Japan

**Keywords:** ultrarapid PCR, *EGFR* mutation, point-of-care testing, virtual bronchoscopic navigation system, endobronchial ultrasonography using a guide sheath

## Abstract

Epidermal growth factor receptor (*EGFR*) gene mutation testing is essential for choosing appropriate treatment options in patients with advanced non-small cell lung cancer (NSCLC). However, a time delay occurs between histological diagnosis and molecular diagnosis in clinical situations. To minimize this delay, we developed a novel point-of-care test for *EGFR* mutations, based on a high-speed real-time polymerase chain reaction (PCR) system designated here as ultrarapid PCR combined with highly accurate bronchoscopic sampling. We investigated whether our system for detecting *EGFR* mutations was valid by comparing test results with those obtained using a commercialized *EGFR* mutation test. We obtained small amounts of bronchial lavage fluids after transbronchial biopsies (TBBs) were performed on enrolled patients (n=168) who underwent endobronchial ultrasonography using a guide sheath (EBUS-GS). *EGFR* mutation analysis was performed by ultrarapid PCR immediately after EBUS-GS-TBBs were obtained (on the same day). After pathological diagnoses of NSCLC, *EGFR* mutation status in formalin-fixed, paraffin- embedded samples was confirmed by the PCR-invader method, and the concordance rates between the PCR methods were compared. The total diagnostic yield of EBUS-GS-TBB was 91.0%. The positive concordance rates for detecting 19del and L858R with the ultrarapid PCR and PCR-invader methods were both 100%. Negative concordance rates were 97.2 and 98.1%, respectively. We also demonstrated a dramatic effect of early erlotinib administration, based on ultrarapid PCR results, for a 52-year-old woman suffering from respiratory failure due to severe intrapulmonary metastases with poor performance status. In conclusion, ultrarapid PCR combined with EBUS-GS-TBB enabled rapid and reliable point-of-care testing for *EGFR* mutations.

## Introduction

Over the last decade, the discovery of epidermal growth factor receptor (*EGFR*) gene mutations and the development of tyrosine kinase inhibitors (TKIs) have dramatically changed the treatment strategies for patients with advanced non-small cell lung cancer (NSCLC) (1–5). Therefore, *EGFR* mutation testing is essential for optimal treatment selection for advanced NSCLC patients. Several methods for detecting *EGFR* mutations mainly in formalin-fixed, paraffin-embedded (FFPE) samples have already been validated and applied in practice (6–11). However, these methods adopt relatively complex polymerase chain reaction (PCR) technologies with pre-designed fluorogenic probes, are packaged by manufacturers, and are often available through outside reference laboratories at relatively high rates. In Japan, the use of EGFR-TKIs for chemo-naïve patients has been limited to those with *EGFR* mutations since 2011. Despite this regulation, the majority of community and university hospitals still depend on outside laboratories for *EGFR* mutation testing. Accordingly, there is a time delay between histological diagnosis and molecular diagnosis in clinical situations. In general, obtaining PCR-based *EGFR* test results from outside laboratories requires 7–14 days after tumor sampling. In cases where immediate treatment is critical, failure to provide appropriate molecular targeted therapy due to delayed molecular diagnostic test results may cause fatal outcomes. Therefore, a quicker, simpler, and less expensive point-of-care *EGFR* mutation testing system is needed.

In the field of infectious diseases, a more rapid real-time PCR system for detecting pathogens has been developed (12). Similarly, we have developed a new, simple, high-speed real-time PCR system (referred to as ultrarapid PCR) for the detection of the 2 most common *EGFR* mutations. This assay involves a pair of mutation-specific primers used in combination with a newly developed PCR machine that is equipped with a novel thermo-control mechanism that makes ultrarapid PCR cycling possible.

In-frame deletion in exon 19 (E746-A750del) and the point mutation replacing leucine with arginine at codon 858 of exon 21 (L858R) represent >90% of oncogenic *EGFR* mutations. Large clinical trials have been conducted to establish the efficacy of EGFR-TKIs in targeting the resulting mutated EGFR proteins (1–5). Therefore, we designed a deletion-specific primer targeting the exon 19 E746-A750del mutation and a point mutation-specific primer for the exon 21 L858R mutation. PCR conditions were optimized for amplifying templates harboring each mutation.

Endobronchial ultrasonography using a guide sheath (EBUS-GS) combined with a virtual bronchoscopic navigation system (VBN) is very useful approach for collecting samples from peripheral pulmonary lesions (13–20). However, a major disadvantage of EBUS-GS is the low sample volume that can be obtained, leading to reduced sensitivity in molecular testing. Therefore, we performed this validation study to determine whether ultrarapid PCR can detect *EGFR* mutations with liquid bronchial lavage fluid (BLF) samples after EBUS-GS-transbronchial biopsies (EBUS-GS-TBBs) were taken.

## Materials and methods

### Patients and samples

**Figure 1:**
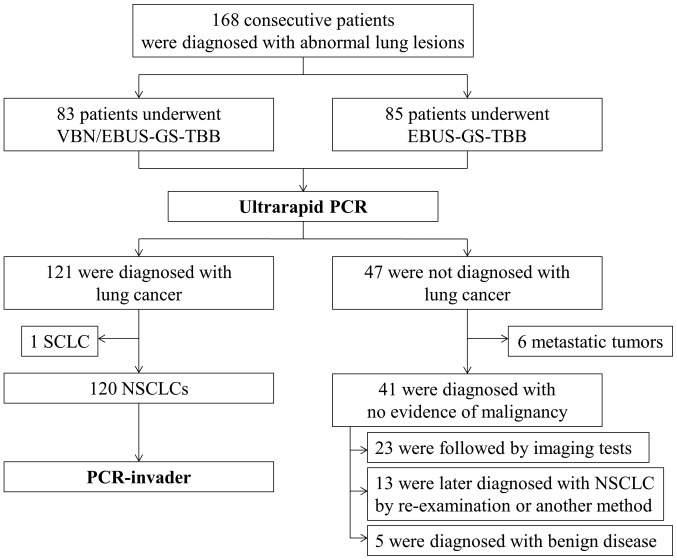
Flow diagram. One or more expert bronchoscopists determined whether to combine EBUS-GS with VBN, based on CT findings. For all 168 patients, analysis of *EGFR* mutations was performed by ultrarapid PCR immediately after the EBUS-GS-TBB procedure. A total of 121 patients (72%) were diagnosed with lung cancer by EBUS-GS-TBB. After a pathological diagnosis of NSCLC was made, *EGFR* mutation status was confirmed by the PCR-invader method. Thirteen patients (8%) who had not been diagnosed with NSCLC by EBUS-GS-TBB were later diagnosed with NSCLC by re-examination or by another sampling method.

**Figure 2:**
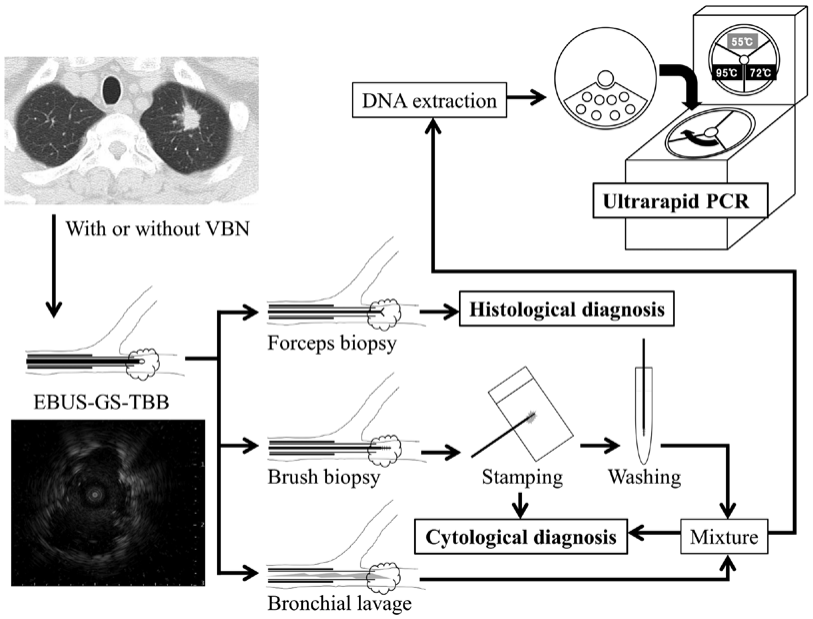
Examination flow chart. An EBUS probe with GS was led into the target lesion and adjusted with EBUS imaging. After removing the EBUS probe, forceps and brush biopsies were performed. At the end of the examinations, bronchial lavages were performed with 20 ml of saline. DNA was extracted from a mixture of bronchial lavage fluid and brush washings.

A total of consecutive 168 patients who underwent EBUS-GS-TBB at the Tottori University Hospital (Yonago, Japan) from November 2012 to December 2013 were enrolled prospectively (Fig. 1). Eligible patients had undiagnosed pulmonary lesions suspected to be lung cancer on chest computed tomography (CT) findings. Samples were prepared by mixing BLFs obtained during EBUS-GS-TBB procedures with saline solutions mixed with EBUS-GS-brush biopsy samples after they were stamped on glass slides. DNA was extracted from patient fluid samples using the QIAamp Blood Mini kit (Qiagen, Tokyo, Japan) (Fig. 2).

Ethical approval was obtained from the Tottori University Hospital and informed consent was obtained from all patients involved prior to performing bronchoscopies.

### VBN and EBUS-GS-TBB procedures

VBNs were performed following approval from physicians and expert bronchoscopists, based on CT findings. CT scan data from multi-detector chest CTs (64- or 128-row; slice width, 0.5 mm) were acquired from all patients before EBUS-GS-TBB. Individual CT data sets from VBN/EBUS-GS group were transferred to a workstation on which VBN software (Bf-NAVI; Cybernet Systems, Co., Ltd., Tokyo, Japan) automatically created VBN images within 15 min. VBN images could be moved multi-directionally on a monitor beside the video-bronchoscopic monitor. All patients were anaesthetized with midazolam and examined using a P260F video bronchoscope (4.0 mm outer diameter; Olympus Corp., Tokyo, Japan). The bronchoscope was introduced into the targeted bronchus with VBN support or the guidance of 2 expert bronchoscopists based on CT axial images. Peripheral target lesions were visualized using a 20 MHz radial-type EBUS probe (external diameter, 1.4 mm; UM-S20-17S; Olympus) with a GS (K-201; Olympus) through a working channel. Ultrasound images were processed in an ultrasound scanner (EU-ME-1 or EU-ME2; Olympus). Pathological samples were collected using forceps and brushes through the GS. Biopsy samples were immediately fixed in formalin. After biopsies were obtained, the target area was washed with 20 ml of saline.

### Mutation-specific PCR using an ultrarapid PCR machine

*EGFR* exon 19 E746-A750 deletion type 1 (2235-2249del; 5′-GGAATTAAGAGAAGC-3′) and exon 21 L858R (2573T>G) were detected using a novel high-speed real-time PCR machine, namely a Hyper-PCR UR104MK IV (Trust Medical Co., Ltd., Kasai, Japan), with allele-specific primers and SpeedSTAR HS DNA Polymerase (Takara Bio, Inc., Shiga, Japan). The UR104MK IV PCR machine utilized a novel temperature control technology. In this system, the PCR mixture is enclosed in a small vessel on a thin, flexible plastic disk and sealed with adhesive film, and the disk is rotated rapidly onto 3 separated heat elements. Rapid PCR can be accomplished by controlling the speed of rotation and the temperature of the 3 heat elements. The UR104MK also has the capacity for real-time monitoring of PCR reactions with a fluorescent probe and post-PCR melt curve analysis. The typical time for amplification and detection when using this machine was <10 min.

Optimized reaction mixtures contained 1.6 μl of 10X Fast buffer I (Takara), 1.3 μl of 2.5 mM dNTPs, 0.4 μl of each allele-specific primer (10 μM), 0.2 μl of SpeedSTAR HS DNA Polymerase (5 U/μl; Takara), 1 μl of template DNA, 1.6 μl of 1:2,000 SYBR-Green, and 9.5 μl of ddH_2_O in a volume of 16 μl. Furthermore, dimethylsulfoxide was added to obtain a final concentration of 5%. PCR thermal cycling conditions were as follows. To amplify E746-A750del type 1, we used 1 cycle of 94°C for 1 min, followed by 35 cycles of 98°C for 1.3 sec, 55°C for 5 sec, and 72°C for 3 sec. To amplify DNA sequences harboring the L858R point mutation, we used 1 cycle of 94°C for 1 min, followed by 30 cycles of 98°C for 1.3 sec, 68°C for 8 sec and 68°C for 8 sec.

### Sensitivity assay

To validate the sensitivity of the PCR system, sensitivity assays were performed using DNA mixtures extracted from the following cell lines: PC9 (2235-2249del), H1975 (2573T>G) and N417 (wild-type). The PC9 cell line was obtained from the RIKEN Cell Bank (Tsukuba, Japan). The H1975 cell line was obtained from the American Type Culture Collection (Rockville, MD, USA). The N417 cell line was provided by Dr A.F. Gazdar and Dr H. Oie (National Cancer Institute-Navy Medical Oncology Branch, Bethesda, MD, USA). These cell lines were mixed in different ratios. Specifically, the PC9 and N417 cell lines were mixed in ratios of 1:0, 0.5:0.5, 0.1:0.9, 0.01:0.99 and 0:1, respectively, while the H1975 and N417 were mixed in ratios of 1:0, 0.5:0.5, 0.1:0.9, 0.01:0.99 and 0:1, respectively. Analysis of *EGFR* mutations was performed as described above.

### Comparison of ultrarapid PCR with the PCR-invader method

*EGFR* mutation analysis was performed with BLF samples from all 168 patients, regardless of pathological diagnosis, by ultrarapid PCR immediately after EBUS-GS-TBB. After pathological diagnosis of NSCLC, the associated *EGFR* mutation statuses in FFPE samples were evaluated by the PCR-invader method (BML, Inc., Tokyo, Japan), which is used in clinical practice at our hospital. To assess the performance of ultrarapid PCR, we evaluated the concordance rates and calculated kappa coefficients for both the ultrarapid PCR and PCR-invader methods.

### Statistical analysis

Average target lesion diameters and diagnostic yields were calculated for the VBN/EBUS-GS and EBUS-GS groups, respectively, and analyzed using the Mann-Whitney U test and the Chi-squared test between these 2 groups. All P-values were 2-sided. A P-value of <0.05 indicated statistical significance. Concordance rates and Cohen’s kappa coefficients were determined between the ultrarapid PCR and PCR-invader methods. Cohen’s kappa coefficient was calculated as kappa = (Po-Pe)/(1-Pe), where Po is the observed concordance rate and Pe is the expected probability of chance agreement (21). A kappa of zero means that there is no agreement beyond chance, and a kappa of 1.00 means that there is perfect agreement. Values ranging from 0.81 to 1.00 indicate near perfect agreements (22). All data were statistically analyzed using IBM SPSS Statistics, ver. 22.

## Results

### Sensitivity

**Figure 3:**
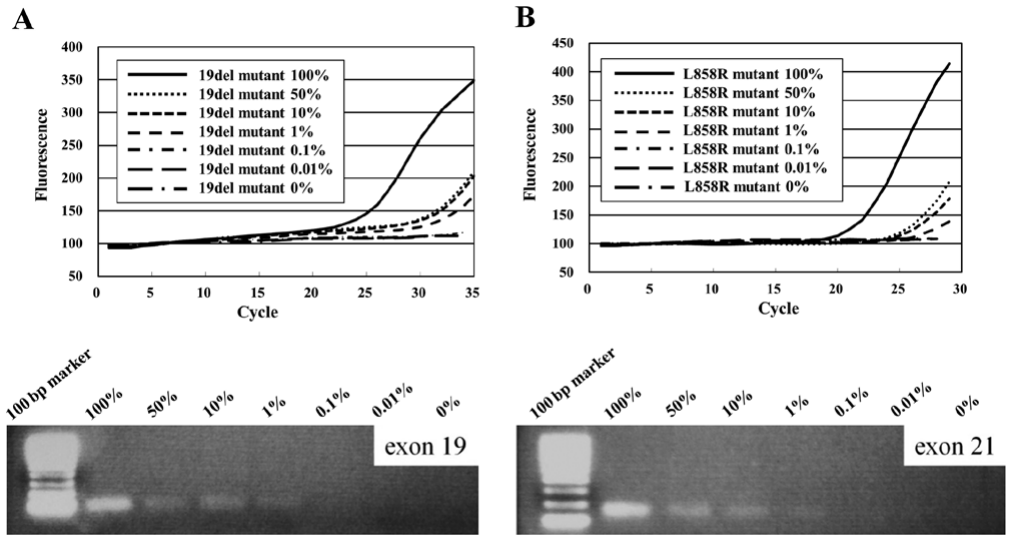
Sensitivity of ultrarapid PCR. (A) Amplification of the 19del allele by ultrarapid PCR was performed using cell samples containing 100, 50, 10, 1, 0.1, 0.01 and 0% PC14 cells, mixed with N417 cells containing 2 copies of the wild-type *EGFR* gene. As few as 1% of tumor cells with the 19del mutation could be detected. (B) Amplification of the L858R allele by ultrarapid PCR using cell samples containing 100, 50, 10, 1, 0.1, 0.01 and 0% H1975 cells, mixed with N417 cells. As few as 1% of tumor cells with L858R mutation could be detected.

The E746-A750del mutation was detected in mixed cell populations containing decreasing percentages (100-1%) of the E746-A750del-positive cell line (PC9) and increasing percentages of the N417 cell line containing 2 copies of the wild-type *EGFR* gene. Similarly, the L858R mutation was detected in cell line mixtures containing 100-1% of an L858R mutation-positive cell line (H1975) and N417 cells (Fig. 3).

### Characteristics of patients and patient samples

VBN was combined with EBUS-GS for 83 out of the 168 patients enrolled in the present study. The median and average diameters of the target lesions were 25 and 30.6 mm, respectively (range, 8–150 mm). In the VBN/EBUS-GS group, the median and average diameters of target lesions were 19 and 20.5 mm, respectively (range, 8–54 mm). In the EBUS-GS group, the median and average diameters of target lesions were 34.5 and 38.6 mm, respectively (range, 8–150 mm; Table I). As shown in Fig. 1, lung cancer was diagnosed histologically in 121 patients, but not in 47 patients, including 5 patients with benign diseases and 6 patients with metastatic tumors. Thirteen out of the 41 patients who were not diagnosed with NSCLC using EBUS-GS-TBB specimens were later diagnosed with NSCLC by re-examination or using another sampling method. Twenty-three patients were provided follow-up with imaging examinations at fixed intervals, and did not show enlargement of peripheral small lesions after EBUS-GS-TBB. After these 23 patients were excluded from the analysis, the total diagnostic yield obtained with EBUS-GS-TBB samples was 91.0% (132/145 cases). In the EBUS-GS-TBB group, the diagnostic yield was 94.6% (70/74 cases), while the diagnostic yield of the VBN/EBUS-GS-TBB was 87.3% (62/71 cases; Table I). Although target lesion diameters were significantly different (P<0.001; Mann-Whitney U test), diagnostic yields were similar in the 2 groups (P=0.18; Chi-squared test).

The median age of the 121 lung cancer patients was 70 years (range, 37–97), and all of the patients were Japanese. NSCLC specimens were classified histologically as adeno-carcinoma in 89 patients (73.6%), squamous cell carcinoma in 22 patients (18.2%), large-cell neuroendocrine carcinoma (LCNEC) in 4 patients (3.3%), adenosquamous carcinoma in 2 patients (1.7%), large cell carcinoma in 2 patients (1.7%), small cell carcinoma in 1 patient (0.8%), and pleomorphic carcinoma in 1 patient (0.8%). The distribution of clinical stages at the time of diagnosis was as follows: 60 patients (49.6%) had stage I carcinoma, 13 patients (10.7%) had stage II, 15 patients (12.4%) had stage III, and 32 patients (26.4%) had stage IV. In 1 patient, the clinical stage was not classified (Table II).

### EGFR mutation detection by ultrarapid PCR

*EGFR* mutations in BLF samples were detected by ultrarapid PCR in 26 adenocarcinoma patients among the 120 NSCLC patients tested (21.7%), but were not detected in any of the 48 patients who were not diagnosed bronchoscopically with NSCLC. Eleven patients (42.3%) had an *EGFR* 19del mutation, and 15 patients (57.7%) had an L858R *EGFR* point mutation (Table III).

### Comparison of the ultrarapid PCR and PCR-invader detection methods

*EGFR* mutations in FFPE tissues were detected in 36 adenocarcinoma patients among 120 NSCLC patients (30.0%) by the PCR-invader method (Table III). Two of these patients (5.6%) had an exon 18 G719A point mutation, 1 patient (2.8%) had a G719C point mutation and an exon 20 S768I point mutation, 1 patient (2.8%) had a G719S and a S768I mutation, 1 patient (2.8%) had a G719C mutation and an exon 21 L858R mutation, 8 patients (22.2%) had an E746-A750del type 1 mutation, 1 patient (2.8%) had an E746-A750del type 2 mutation, 6 patients (16.7%) had low-frequency mutations in exon 19, and 16 patients (44.4%) had an L858R mutation.

As shown in Table IV, positive concordance rates of 19del and L858R between ultrarapid PCR and PCR-invader were both 100%, while negative concordance rates were 97.2 and 98.1%, respectively. The kappa coefficients for detecting the 19del and L858R mutations between ultrarapid PCR and PCR-invader were 0.87 and 0.93, respectively. The average turnaround time for ultrarapid PCR was only 90 min, whereas that for the PCR-invader method by an outside laboratory was 9 days.

### Case report

**Figure 4:**
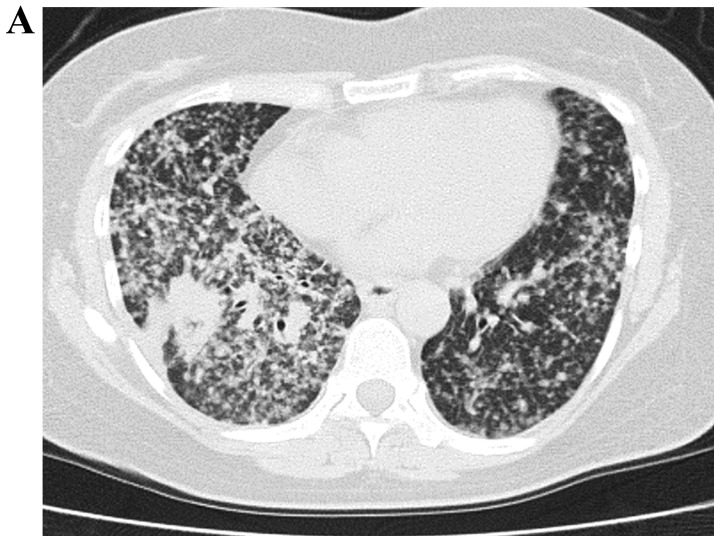

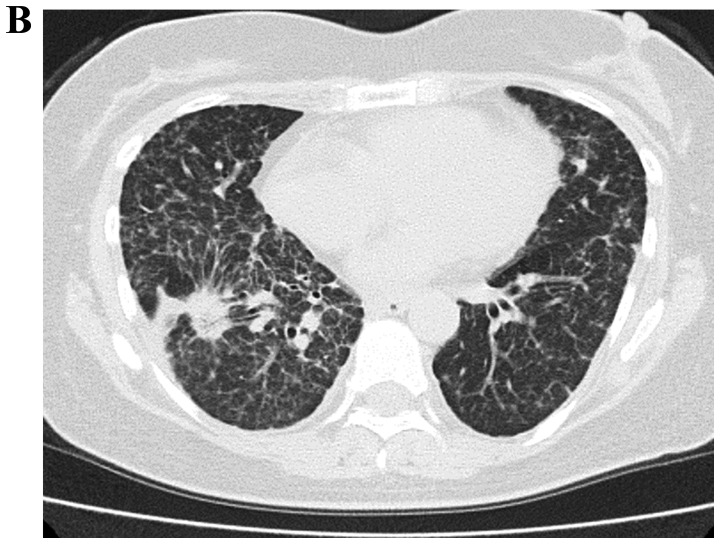
Dramatic effect of EGFR-TKI for a poor PS *EGFR* mutant. A chest CT scan obtained before treatment (A) and at 2 weeks after the administration of erlotinib (B) are shown. The diffuse granular shadow of the bilateral lung field had mostly disappeared after the initiation of therapy. Consequently, the patient’s PS score improved from 3 to 1.

A 52-year-old non-smoking female, without previous illness, was admitted to our hospital because of a dry cough and dyspnea at rest. Her performance status (PS) was 3 on admission. Her chest CT scan showed numerous bilateral diffuse granular lung shadows and a 20 mm-diameter nodular shadow on the lower right lobe (Fig. 4A). Whole body bone scintigraphy was performed later, revealing an abnormal accumulation in the fifth lumbar vertebra. Suspecting that she had advanced lung cancer, we immediately performed an EBUS-GS-TBB against the primary lesion of the lower right lobe. By 60 min after performing the EBUS-GS-TBB procedure, we obtained a positive result for the E746-A750del mutation by ultrarapid PCR. Because she had respiratory failure and a poor PS on admission, she was not eligible for cytotoxic chemotherapy. Therefore, it was deemed appropriate to initiate EGFR-TKI therapy as soon as possible. The following day, we started EGFR-TKI therapy (erlotinib 150 mg orally every 24 h), after obtaining a definitive pathological diagnosis of adenocarcinoma by an immunohistochemical method. Two weeks later, the diffuse and numerous granular shadows of bilateral lung field had mostly disappeared (Fig. 4B). Moreover, her respiratory failure and poor PS score were dramatically improved before PCR-invader results were provided.

## Discussion

Bronchoscopy has been used to diagnose abnormal lung lesions for ~60 years. In recent years, the development of new diagnostic tools, such as EBUS, GS and VBN, has substantially improved diagnostic accuracy. Eberhardt *et al* (15) reported that the combination of EBUS and VBN improved the diagnostic yield in peripheral lung lesions, and VBN/EBUS is recommended for the diagnosis of lung peripheral lesions in guidelines of the European Society for Medical Oncology (23). Ishida *et al* (24) reported that the diagnostic yield of VBN combined EBUS-GS with small peripheral lesions (diameter <30 mm) was 80%. Similarly, we found high diagnostic yields in the present study despite the fact that most target lesions were small, especially in the VBN/EBUS-GS group. The appropriate decisions made regarding whether VBN should be used reinforced the diagnostic accuracy of EBUS-GS-TBBs for small peripheral lesions. Moreover, we usually collect at least 6 or more tissue samples. An advantage of EBUS-GS-TBB is that it is easy to obtain multiple biopsies through the fixed GS safely.

In this study, we validated ultrarapid PCR as a method for detecting the 2 most common *EGFR* mutations in liquid samples obtained by the EBUS-GS-TBB method. In many cases, even though these samples contain a very small amount of tumor cells, our method can detect the major *EGFR* mutations. Previous studies have shown similar results by molecular analysis of liquid samples collected by bronchoscopy. Yamaguchi *et al* (25) concluded that the analysis of *EGFR*, *KRAS* and *TP53* mutations using curette lavage fluids obtained by bronchoscopy was possible. Furthermore, some reports have described the molecular analysis of lymph node samples obtained by EBUS guided trans-bronchial needle aspiration (26–28) or trans-esophageal ultrasound scanning with fine needle aspiration (29,30). Likewise, Buttitta *et al* (31) reported that *EGFR* mutation analysis of bronchoalveolar lavage by next-generation sequencing was possible even in cases where conventional methods failed. Importantly, the accuracy of our method was remarkably high, although the BLF samples contained a small amount of tumor cells.

The greatest advantage of the ultrarapid PCR method is its speed. To the best of our knowledge, ultrarapid PCR is the fastest PCR system for detecting *EGFR* mutations at present. Ultrarapid PCR is completed within 10 min, while other methods take a few hours to detect mutations. This advantage can potentially have positive effects on treatment outcomes in cases requiring urgent treatment by early EGFR-TKI administration. Generally, the administration of cytotoxic agents for patients with poor PS is not recommended (32). However, some reports indicate that the use of EGFR-TKIs in patients with poor PS is effective and feasible because of their relatively mild toxicities (33). It is necessary to be careful in selecting therapeutic measures because TKIs are associated with an increased risk for developing interstitial pneumonitis in patients with poor PS scores (34). In addition, it will also be important to explore therapeutic opportunities for improving prognoses.

Most *EGFR* mutations are located in exon 18, 19, 20 and 21, with ~90% of these mutations occurring in exons 19 and 21 (35). In previous phase III trials with EGFR-TKIs, patients with hotspot mutations (exon 19 deletions or exon 21 L858R) were mostly recruited. The response rate of patients with these hotspot mutations was ~80% (2,5). In contrast, the response rate of patients with minor mutations, such as exon 18 point mutation G719X and exon 21 point mutation L861Q, was only 20% (36). Moreover, EGFR-TKIs had no proven survival benefit in patients with minor mutations (36). Therefore, we limited our search to these hotspot mutations in this study.

As demonstrated in our case report, ultrarapid PCR can deliver quick results in practical clinical situations. Patients with hotspot mutations in need of immediate care should receive EGFR-TKI treatment as soon as possible. Failures in providing appropriate molecular therapy due to molecular diagnosis delays should be avoided.

Despite the promising results obtained using ultrarapid PCR for detecting major *EGFR* mutations, a limitation of this method is that it can only detect known mutations. Detecting minor *EGFR* mutations in exon 18 and the T790M point mutation associated with drug resistance (exon 20) will require the development of additional probes. This current limitation reduces patients’ opportunities for rapid qualification for the third-generation EGFR-TKIs therapy, such as AZD9291 (37) by ultrarapid PCR alone. However, this problem may be solved by the development of additional primer sets for minor mutations in the near future.

In conclusion, it was demonstrated that ultrarapid PCR is an extremely quick and precise method for examining clinical liquid samples with a background of normal cells. The combination of ultrarapid PCR and EBUS-GS-TBB methods may enable point-of-care testing for NSCLC patient samples harboring *EGFR* mutations.

## Figures and Tables

**Table I:** Comparison of target lesions diameters and diagnostic yields between VBN/EBUS-GS-TBB and EBUS-GS-TBB.

	VBN/EBUS-GS-TBB (N=83)	EBUS-GS-TBB (N=85)	P-value
Diameter (mm)			
Median	19.0	34.5	
Average	20.5	38.6	<0.001^b^
Range	8–54	8–150	
Diagnostic yield^a^	87.3% (62/71 cases)	94.6% (70/74 cases)	0.18^c^

a The diagnostic yield of EBUS-GS-TBB was 91.0% (132/145). The diagnostic yield was calculated for all patients, except for 23 patients that were provided follow-up with imaging examinations at fixed intervals and for whom enlargement of peripheral small lesions after EBUS-GS-TBB was not observed.

b Mann-Whitney U test;

c Chi-squared test.

**Table II:** Patient characteristics.

Characteristics	Diagnosed with lung cancer by EBUS-GS-TBB^a^ (N=121)	Not diagnosed with lung cancer by EBUS-GS-TBB (N=47)
Age (years)		
Median	70	71
Range	37–97	65–87
Male gender, n (%)	75 (64.1)	29 (56.9)
Smoking status, n (%)		
Current smoker	34 (28.1)	7 (14.9)
Former smoker	48 (39.7)	22 (46.8)
Never smoker	39 (32.2)	18 (38.3)
Histologic type, n (%)		
Adenocarcinoma	89 (73.6)	
Squamous cell carcinoma	22 (18.2)	
Large cell carcinoma	2 (1.7)	
Small cell carcinoma	1 (0.8)	
Adenosquamous carcinoma	2 (1.7)	
LCNEC	4 (3.3)	
Pleomorphic	1 (0.8)	
Stage, n (%)		
I	60 (49.6)	
II	13 (10.7)	
III	15 (12.4)	
IV	32 (26.4)	
Not evaluated	1 (0.8)	

a A total of 121 patients were diagnosed bronchoscopically with lung cancer. Out of 121 cancers, 89 (73.6%) were adenocarcinoma and 32 (26.4%) were stage IV.

**Table III:** Comparison of ultrarapid PCR and PCR-invader test results found when detecting the 2 most common *EGFR* mutations in samples from 120 NSCLC patients.

	PCR-invader		
	
Ultrarapid PCR	Mutation (+)	Mutation (−)	Total
19del			
Mutation (+)	11	0	11
Mutation (−)	3	106	109
Total	14	106	120
L858R			
Mutation (+)	15	0	15
Mutation (−)	2	103	105
Total	17	103	120

**Table IV:** Concordance rates and Cohen’s kappa coefficients between the ultrarapid PCR and PCR-invader methods.

Concordance rate	19del (%)	L858 (%)
Positive	100	100
Negative	97.2	98.1
Kappa coefficient^a^	0.87	0.93

a A range from 0.81 to 1.00 corresponds to near perfect agreement.
